# Development of ^222^Rn Emanation Sources with Integrated Quasi 2π Active Monitoring

**DOI:** 10.3390/ijerph19020840

**Published:** 2022-01-12

**Authors:** Florian Mertes, Stefan Röttger, Annette Röttger

**Affiliations:** Physikalisch-Technische Bundesanstalt, National Metrology Institute, 38116 Braunschweig, Germany; stefan.roettger@ptb.de (S.R.); annette.roettger@ptb.de (A.R.)

**Keywords:** ^222^Rn emanation, physical vapor deposition, silicon detectors

## Abstract

In this work, a novel approach for the standardization of low-level ^222^Rn emanation is presented. The technique is based on the integration of a ^222^Rn source, directly, with an α-particle detector, which allows the residual ^222^Rn to be continuously monitored. Preparation of the device entails thermal physical vapor deposition of ^226^RaCl_2_ directly onto the surface of a commercially available ion implanted Si-diode detector, resulting in a thin-layer geometry. This enables continuous collection of well resolved α-particle spectra of the nuclei, decaying within the deposited layer, with a detection efficiency of approximately 0.5 in a quasi 2π geometry. The continuously sampled α-particle spectra are used to derive the emanation by statistical inversion. It is possible to achieve this with high temporal resolution due to the small background and the high counting efficiency of the presented technique. The emanation derived in this way exhibits a dependence on the relative humidity of up to 15% in the range from 20% rH to 90% rH. Traceability to the SI is provided by employing defined solid-angle α-particle spectrometry to characterize the counting efficiency of the modified detectors. The presented technique is demonstrated to apply to a range covering the release of at least 1 to 210 ^222^Rn atoms per second, and it results in SI-traceable emanation values with a combined standard uncertainty not exceeding 2%. This provides a pathway for the realization of reference atmospheres covering typical environmental ^222^Rn levels and thus drastically improves the realization and the dissemination of the derived unit of the activity concentration concerning ^222^Rn in air.

## 1. Introduction

### 1.1. Background and Motivation

^222^Rn is a naturally occurring radioactive noble gas, generated in the decay chain of primordial ^238^U, and is thus released from soil to the atmosphere through diffusion processes. ^222^Rn can be accumulated inside buildings and has been estimated to be the second most important cause of lung cancer. It is also the most relevant contributor to the average effective dose from natural sources experienced by the general public [1,2,3], which is why radon measurements are of interest for public health, radiation protection, and associated legislation. Moreover, in the environmental sciences, ^222^Rn, in ambient air, was found to be an interesting proxy for mixing processes and terrestrial influence, so the measurement of its concentration finds a multitude of applications [4,5,6,7,8,9,10,11,12,13]. Activity concentrations of ^222^Rn in outdoor air are in the order of a few Bq⋅m^−3^. The implementation of large-scale ^222^Rn monitoring networks, to provide concentration data for the environmental sciences, and ensuring their comparability requires calibration techniques, for radon monitors in this concentration range, that are traceable to the international system of units (SI), as addressed in the project 19ENV01 traceRadon [14].

For this purpose, it will be necessary to realize and disseminate the unit Bq⋅m^−3^ for ^222^Rn in air, with a small uncertainty over the required range, in a way that is traceable to the SI. For calibrations at such remarkably low activity concentrations, decaying reference atmospheres of ^222^Rn, e.g., produced by the method of Picolo et al. [15], are generally unsuitable for statistical reasons. Relatively recently, an alternative was found in the use of so-called ^222^Rn emanation sources [16,17,18], which are ^226^Ra sources constructed with such physicochemical properties that a known or measurable amount of ^222^Rn is released per unit time, which enables calibration at static or even dynamic activity concentrations. Since the processes resulting in this release are generally linked to the physicochemical properties of the source material, the ^222^Rn emanation from such sources must be expected to vary with environmental parameters such as humidity, temperature, and pressure. The correlation of emanation with these parameters has previously been reported for a variety of different materials, e.g., in [19,20,21]. It is, therefore, of interest to construct ^222^Rn sources whose emanation can be monitored during operation to account for these factors, especially in the case of in-field calibrations of ambient level ^222^Rn monitors, where the exact control of all relevant climatic parameters is not feasible. In the following we present a new method of construction of such sources, specifically designed to overcome the challenges that result from the considerably low activity range of ^226^Ra that is needed to realize reference atmospheres in the outdoor concentration range by combining the ^222^Rn source and detector into one system. The full traceability chain to the SI, regarding determination of the ^222^Rn delivery by this system, is laid down, along with a discussion of possible causes for systematic bias and the limits of the presented technique. Additionally, a data analysis method, enabling the estimation of near real-time values of ^222^Rn released from the system (in terms of atoms per unit time) through statistical inversion, is briefly presented, which is tightly coupled with its design and is applied to provide emanation estimates in times of non-steady-state situations.

### 1.2. Theoretical Considerations for ^222^Rn Emanation Standards for Outdoor Activity Concentrations

Generally, state-of-the-art methods to quantify the ^222^Rn emanation from solid sources (in a primary way, i.e., not using ^222^Rn concentration measurement devices that require calibration) rely on measuring activity ratios of ^226^Ra and the residual γ-ray emitting ^222^Rn progeny, ^214^Pb and ^214^Bi, inside an emanation source to deduce the steady-state release of ^222^Rn or, equivalently, a partitioning coefficient of ^222^Rn between the free volume and the source volume [16,18,22]. The basis of this method is the conservation of the total amount of ^222^Rn nuclei that are generated by the source, which can either emanate from the source or decay within the source, which is expressed by the following first order kinetics.

(1) $$\frac{dA_{Rn-222}}{dt}=-\lambda_{Rn-222}A_{Rn-222}+\lambda_{Rn-222}A_{Ra-226}-\lambda_{Rn-222}\eta$$

where η describes the number of released ^222^Rn atoms per unit time and A denote the activities of the respective nuclides in the emanation source.

Therefore, time-resolved measurements of the ^222^Rn and ^226^Ra activity of an emanation source allows one to estimate η, e.g., trivially in the steady-state of $\frac{dA_{Rn-222}}{dt}=0$.

However, at the low activities needed to realize reference atmospheres at the ambient levels, γ-ray measurements of the equilibrated ^222^Rn progeny ^214^Pb and ^214^Bi are not readily efficient enough to provide good temporal resolution of this method, especially regarding the real-time monitoring and considering the ubiquitous Poisson noise. A more direct, and much more sensitive, method entails the direct measurement of ^222^Rn that remains in the emanation source, henceforth referred to as residual ^222^Rn, through detection of its α-particles. However, with conventional α-particle spectrometry techniques, this is only possible in vacuo, e.g., [23], and thus, it is generally not useful to investigate emanation behavior directly under ambient conditions. This is due to the rapid energy loss of α-particles in any type of material, which leads to the significant distortion of α-particles spectra, recorded at a finite distance between the source and the detector, at ambient pressure. In such a spectrum, the contributions of ^226^Ra and ^222^Rn would no longer be well resolved, at which point the measurement of residual ^222^Rn is not reliable.

The main aim of this work is to establish a method to use α-particle spectrometry to realize the supporting measurements needed to apply Equation (1) for standardization of η, which will allow for a reduction of statistical uncertainties associated with the inference of η and hence, to realize reference atmospheres at the ambient levels, even at changing environmental conditions.

**Figure 1:**
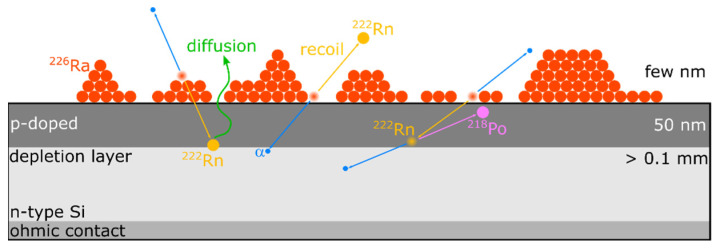
Schematic of silicon detector, modified with a thin layer of ^226^Ra (IRSD). The silicon detector is made up of n-type silicon and a p-doped front side contact. Reverse biasing of the p/n-junction results in a depletion layer of several 0.1 mm thickness, which is the sensitive detection volume. The ^222^Rn emanation mechanisms of recoiling and diffusion are depicted, as well as the spatial displacement of progeny along the decay chain. Details are given in the text.

One way to realize the direct α-particle spectrometry of the residual ^222^Rn is to minimize the source-detector distance, i.e., ultimately, by direct construction of the source on, or even within, an α-particle spectrometric detector. Such a setup will henceforth be referred to as the Integrated ^222^Rn Source/Detector (IRSD) and is proposed, discussed, and implemented within this work for the first time. It is schematically depicted in Figure 1. Typically, an α-particle spectrometer, such as the one used for the IRSD, is made up of an n-type silicon wafer that is p-doped at its entrance window, nowadays commonly through ion implantation, which results in entrance windows on the order of 50 nm thickness. To operate such a detector, the resultant p/n-junction is reversely biased from a backside ohmic contact to form a depletion layer of minimal free charge carriers. Due to their high interaction probability with matter, α-particles, which enter this layer of few 0.1 mm in thickness, are detected with practically unity probability, resulting in an electrical impulse that is proportional to the incident α-particle energy. Therefore, the theoretical detection probability in this configuration is 50% resulting from the 2π sr solid-angle subtended by the detector. Moreover, the typical background in α-particle spectrometry is orders of magnitude smaller than in any γ-ray spectrometric setup, and considering that the latter requires bulky lead shielding, emanation sources monitored by α-particle spectrometry are strongly preferred for the realization of in-field calibration procedures. In addition, α-particle spectrometers are typically a factor of 10 to 100 cheaper than γ-ray spectrometers. For these reasons, α-particle spectrometry is the superior choice for the purpose of monitoring the amount of residual ^222^Rn.

However, in the hypothesized configuration, as shown in Figure 1, the IRSD, the p-doped entrance window and the deposited ^226^Ra layer must be as thin as possible to minimize the variance in the energy loss of traversing α-particles. As a result of the variance in the energy loss, peaks in α-particle spectra generally show a left-handed (low energy) tailing that can lead to considerable overlap in the spectra and, thus, to significant difficulty of their analysis. Especially at such an infinitesimally small source-detector distance, the variance in the path length of α-particles entering the depletion layer through absorbing intermediate matter is high. Thus, pronounced low energy tailing of peaks in spectra, recorded in such a configuration results and methods to construct such a device must be chosen considering the mass of deposited impurities. Correspondingly, the analysis of α-particle spectra, which entails the determination of peak areas, must be carried out considering both the significance of the tailing and the specific nuclide composition at hand.

From the necessary thin layer of ^226^Ra for the construction of the IRSD, the ^222^Rn nuclei are released by two distinct processes. After the α-decay of ^226^Ra, the ^222^Rn nucleus carries a recoil energy of 86 keV on average, which is enough to overcome the binding energy of chemical bonds, electrostatic attraction, and other adsorption forces and to penetrate few nm of a solid material and several 10 µm of ambient pressure air [24]. Hence, the generated ^222^Rn is, in part, released directly as a result of this recoil energy. Since the recoiling takes place isotropically, in a random direction, a fraction of up to 50% of the generated ^222^Rn nuclei are implanted into the first few nm of the silicon detector. These ^222^Rn nuclei may be subsequently released through a diffusion process, which supposedly depends strongly on the chemical composition of the radium layer and the temperature. Analogous displacement occurs for ^218^Po and ^214^Po, both of which are nuclei that result directly, and indirectly, from an α-decay within the decay chain of ^226^Ra.

In this work, we investigate the feasibility and resultant performance of an IRSD system, produced by physical vapor deposition of ^226^Ra, onto commercially available Si-detectors. Specific analytical techniques, which are detailed in Section 2, have been developed to best utilize the data that can be obtained by operation of the resultant IRSD to measure and, hence, to standardize the amount of emanating ^222^Rn, even in non-steady state situations.

## 2. Materials and Methods

### 2.1. Construction of ^226^Ra Modified Ion-Implanted Si-Diode Detectors

**Figure 2:**
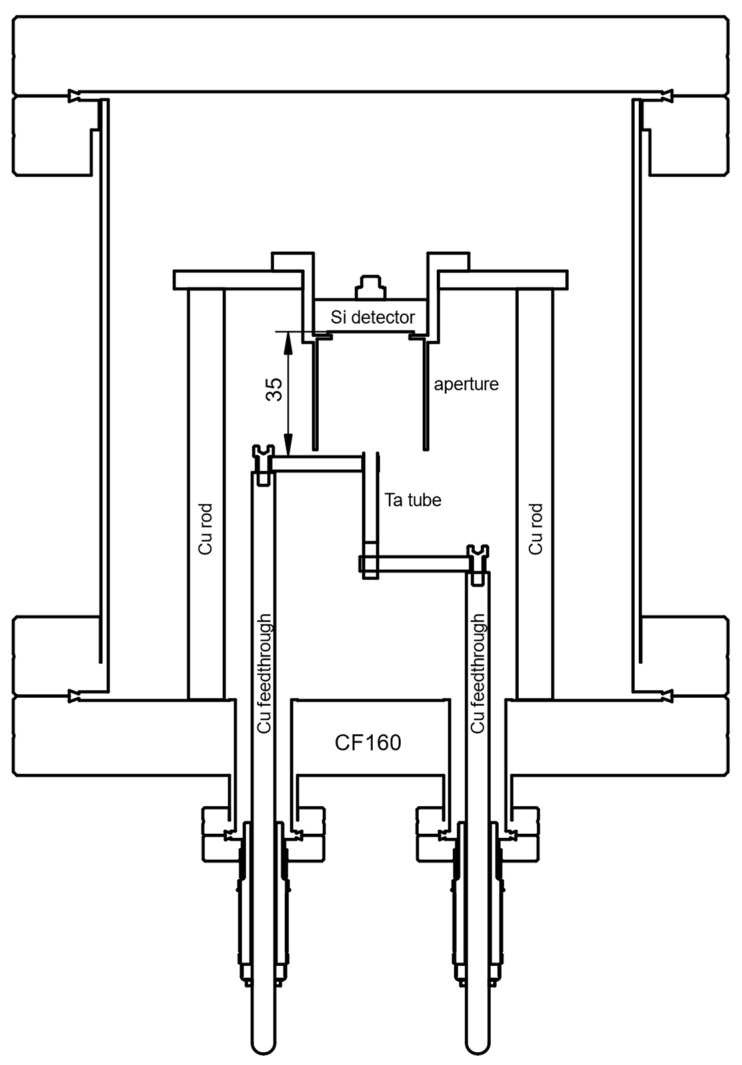
Schematic drawing of the custom thermal-PVD setup used in this work. Drawing not to scale. Details of the setup are described in Section 2.1.

A specifically designed thermal physical vapor deposition (thermal-PVD) unit, depicted schematically in Figure 2, was implemented for the procedure of depositing a ^226^Ra containing thin-layer directly onto commercially available implanted Si-diode detectors (e.g., Mirion PIPS^®^ series, Ametek Ortec ULTRA^®^ series) to implement the IRSD. It was built from standard conflat-flange components (316L stainless steel) with copper seals. The unit is equipped with a sample holder for mounting the detector to be modified with a ^226^Ra layer (the future IRSD) at a nominal distance of 35 mm from the opening of a tantalum tube (EVOCHEM Advanced Materials) of approximately 25 mm length and 4 mm inner diameter. The tantalum tube was heated resistively, using powers up to 120 W DC, estimated (Stefan–Boltzmann law) to roughly correspond to a temperature of 1500 K in steady-state. The sample holder features a stainless-steel aperture system to confine the deposited ^226^Ra layer by shadowing with a diameter of (20.0 ± 0.1) mm to minimize possible edge effects on the approx. 25 mm active diameter of the Si detectors. The aperture is tubular and elongated to the level of the opening of the tantalum tube to avoid contamination of the vacuum chamber as much as possible, presuming molecular flow and line-of-sight deposition. The aperture system was cleaned of ^226^Ra with diluted HCl when the built-up contamination was found to be too large. Chamber pressure was maintained at around 10^−4^ Pa when the unit was operating using a membrane- and a turbomolecular pump (Pfeiffer vacuum HiPace80), while at ambient temperature, pressures as low as 5 × 10^−6^ Pa were attained.

As radium compounds are generally comparable to their barium homologues, it was presumed that RaCl_2_ would show reasonably high vapor pressure at 1300 K, similar to BaCl_2_, with reportedly around 1 Pa to 10 Pa in this range [25,26], making it very feasible to evaporate, or even sublime, this radium compound at pressures in the order of 10^−4^ Pa to 10^−3^ Pa. Supposedly, RaCl_2_ exists as a gas-phase molecule, and therefore, the species deposited using the present method is presumed to be RaCl_2_, which is thought to form its dihydrate upon contact with ambient moisture. Nonetheless, the sub-halide RaF of radium has been reported and investigated for radium recently [27], and the sub-halides are well known to exist for barium in the form of BaF and BaCl, which is why a mixture of radium from decomposition, radium chloride, and radium subchloride might be deposited using this method. Due to the chemical reactivity of some of the deposited species, it is expected that the chemical composition changes upon first exposure to the atmosphere.

As a source of ^226^RaCl_2_ for the outlined thermal-PVD process, a PTB standard solution, nominally 71 kBq RaCl_2_ (^226^RaCl_2_ in 0.1 HCl with 0.5% m/m BaCl_2_), was converted into the nitrate and purified from its Ba^2+^ carrier through extraction chromatography with Sr-Resin^®^ [28] (4,4′(5″)-di-tertbutyl-di-cyclohexano-18-crown-6 in 1-butanol dispersed on SiO_2_-particles). This step was deemed necessary to reduce the amount of BaCl_2_ present and, hence, to minimize the amount of deposited material on the future IRSDs. The chromatography was monitored by the addition of nominally 13 kBq ^133^Ba as a radiotracer. Resultant ^133^Ba-free fractions were pooled and converted back into the chloride by addition, and subsequent evaporation, of conc. HCl and aliquoted for later use. This method was previously used in the production of reduced carrier ^226^Ra solutions in [16], with optimized conditions based on [29]. ICP-MS was used to determine that the residual amount of Ba^2+^ was on the same order as the content of ^226^Ra^2+^ (concerning atom numbers). For each deposition, an aliquot of this solution was transferred to a 0.5 mL conical bottom polyethylene flask, evaporated to dryness, and taken up in a suitably small volume (e.g., 0.1 mL) of 0.5 M HCl (Methanol was also tested, but it was found to lead to considerable losses by wall-attached or undissolved ^226^Ra) to allow for transfer into the tubular tantalum evaporation source in which the solution was allowed to dry. For the removal of crystal water from the resultant ^226^RaCl_2_-dihydrate in the tube, the heating power was maintained at 20 W for the first 30 min of each deposition. Power was subsequently increased to a maximum of 120 W over 20 min. Deposition efficiencies, on the order of 15%, were experienced for this specific setup (including losses from solution transfer), mainly caused by the specific deposition geometry. However, a non-negligible gross-alpha count rate was observed in close proximity to the tantalum tube orifice, likely attributable to the ^226^Ra that did not make its way out of the tube. This might be due to the formation of highly non-volatile tantalates or chloro-tantalates.

A total of four IRSD were constructed in this way, and results are compiled in Table 1, where the deposited ^226^Ra activity and the counting efficiency for ^226^Ra were determined, as presented, in Section 2.4. Before the deposition onto detectors was carried out, bare prime-grade polished 1” p-type Si-wafers were modified with ^226^RaCl_2_ on the order of 10 Bq to be investigated by scanning electron microscopy (SEM, *Thermo Fisher Scientific Verios G4,* through-lens secondary electron detector).

The thin-film production method must be gentle enough not to damage the p/n-junction characteristics of the detector, which could potentially result from knock-ons during ion implantation or sputter deposition, temperature-induced diffusion of junction dopants, or excessive contamination. Thermal stress in the form of rapid or excessive heating and cooling of the detectors, along with the associated force exerted from the expanding housing and backside contact, can easily shatter the Si-wafer.

In spite of potential thermal stress, thermal physical vapor deposition (thermal-PVD) was employed in this study due to its relative simplicity and ability to provide relatively clean deposits, compared with, e.g., electrodeposition.

A tubular geometry was chosen for the vaporization unit, since it was expected to provide a somewhat directional effusion of the emerging gas-phase molecules as a function of the diameter to length ratio, favoring the fraction of ^226^Ra, released into the solid-angle subtended by the future IRSD. This geometry is, thus, presumed to result in increased deposition efficiency at the cost of reduced uniformity.

### 2.2. Operation of Integrated ^222^Rn Sources/Detectors

IRSDs constructed as described above were operated using standard pre-amplifiers (Mirion, Model 2018EB, and Ametek Ortec, Model 142B, respectively) in a light-tight environment at ambient and reduced pressures. Pulse height spectrum acquisition was carried out using a labZy nanoMCA-II and a Mirion Lynx, using integration times between 600 s and 3600 s, adjusted to the ^226^Ra activity of the respective IRSD. The bias voltage was chosen according to the manufacturer’s specifications. For general spectrum acquisition, the humidity and temperature of the environment were not controlled. However, two IRSDs were also operated in a nominal 50 L closed volume in which the temperature, relative humidity, and pressure were recorded. At specific times, the relative humidity in this volume was changed by the introduction of warm water or by flushing with laboratory air to investigate the dependence of the emanation of each IRSD on the relative humidity. In this case, the method described in Section 2.5 was used to calculate the emanation based on time-series of collected α-particle spectra.

### 2.3. Autoradiography

**Figure 3:**
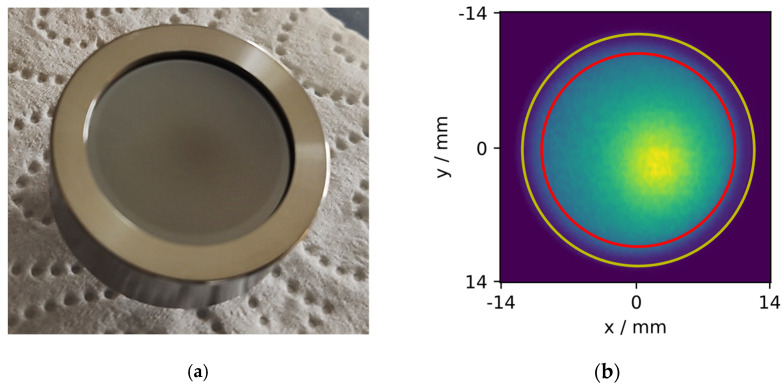
(**a**) shows a photograph of an IRSD based on a 450 mm^2^ Canberra PIPS^®^ detector, modified with a layer containing 440 Bq ^226^RaCl_2_. (**b**) shows a digital autoradiograph obtained from such a deposit where the inner diameter of the recessed Si-surface is given in yellow and the diameter of the shadowing aperture is given in red.

To investigate the spatial distribution of radioactivity on each of the produced IRSDs, autoradiographs were recorded using a FUJIFILM FLA-9000 readout device and digital radiography films. Four ^238^Pu reference point sources and a 3d printed holder were used to place the ^238^Pu sources rectangularly around the respective IRSD. The images of the ^238^Pu sources created on the radiography film were used to position a 140 × 140 grid of (0.2⋅0.2) mm^2^ pixels over which the readout was conducted. The grid was placed in such a way that it was centered with respect to the ^238^Pu sources and, due to the sample holder, also with respect to the outer diameter of the IRSD housing. The IRSDs were placed directly on top of the film, resulting in a displacement of the active surface of the detectors of approximately 1 mm from the radiography film because of the recess in the detector housing (Figure 3a).

### 2.4. Alpha-Particle Spectrometry under Defined Solid-Angle

#### 2.4.1. General Defined Solid-Angle Setup

The PTB primary defined-solid angle (DSA) α-particle spectrometry system was used to perform DSA α-particle spectrometry of the produced IRSDs, with an approximate geometrical efficiency of 1%, as similarly used in [16,23]. DSA α-particle spectrometry is among the most accurate tools for the standardization of activity of α-emitting nuclides, routinely achieving uncertainties below 1% [30,31]. The basis of this method is the calculation of the solid-angle, subtended by an α-particle detector, and hence, its counting efficiency through precise knowledge of the measurement geometry, defined by an aperture system. This absolute measurement technique was used to determine the deposited ^226^Ra activity, traceably to the SI, which was subsequently used to calibrate the counting efficiency of each IRSD by comparison of the IRSD α-particle spectrum with the determined value of its ^226^Ra activity. In this way, traceability to the SI is established.

IRSDs were mounted in the DSA spectrometer using a 3d printed holder and a spring-loaded screw to push the housing against the bottom aperture of the DSA setup. All geometrical parameters of the reference DSA setup are known, traceably, to PTB standards and, with little uncertainty, are given in more detail in [16]. However, the recess depth of the Si-surface in the IRSD was unknown. The effective distance of the active IRSD surface to the top aperture was determined for each measurement using a calibrated, digital depth micrometer screw (Mitutoyo, resolution 0.001 mm), relative to the standard sample holder, of which distance from the top aperture is known. Each depth measurement was repeated for a total of eight positions on the IRSD wafer, results were averaged, and their standard deviation was used as the uncertainty (generally > 0.03 mm) of the source to aperture distance since it was higher than the uncertainty of the micrometer screw measurement, likely due to tilting and/or warping introduced by the wafer mounting mechanism (frontside contact) in the housing and the IRSD holder. The system was operated at chamber pressures of approx. 10^−1^ Pa. Spectra of the reference DSA detector, as well as the IRSDs, were each recorded over integration times that were chosen concerning each IRSD’s ^226^Ra activity. However, spectra obtained were summed up for data analysis, neglecting possible gain-shifts. Total measurement times were adjusted concerning each deposited activity.

#### 2.4.2. Calculation of Geometrical Efficiency

The geometrical efficiency G of the DSA setup was calculated separately for each measurement by evaluating the following relation through Monte–Carlo integration,

(2) $$G=\frac{1}{4\pi }\frac{\int_{A}^{}w_{dA}\Omega_{dA}dA}{\int_{A}^{}w_{dA}dA}$$

where $\Omega_{dA}$ denotes the solid angle of the area element dA subtended by the detector, $w_{dA}$ denotes the relative activity weight of the area element, and A denotes the total source area.

The uncertainty of G was estimated by resampling the geometrical parameters within their experimentally determined probability distributions, similar to [16,32]. As reported therein, the computation of G was carried out for each specific realization of the geometry through tracking randomly generated paths through the geometry and counting the detector hits. The sampling of origin points was carried out by using the experimentally determined, uniformly oversampled activity distribution from the autoradiographs from Section 2.2. A randomly distributed rotational angle uniform in the circle, a normally distributed pixel size uncertainty of 10%, and normally distributed x,y-offsets, with a standard deviation of 0.5 mm each, are included in the analysis of the geometrical uncertainty.

#### 2.4.3. Peak Area Analysis

To derive the activity from α-particle spectra, peak areas are to be determined accurately. As discussed in Section 1.2, the peaks in the α-particle spectra, recorded with the IRSDs, show a slight overlap due to their left-handed tailing. To account for this, the peak areas in all recorded α-particle spectra were determined using non-linear regression based on a refined version of the models introduced in [33] and further improved by [34]. In these modeling procedures, each peak is represented as a mixture of exponentially modified Gaussians (ExGaussian), often with shared tailing parameters.

Due to the slight difference of the distance of each α-emitting nuclide (^226^Ra, ^222^Rn, ^218^Po, and ^214^Po) to the depletion layer (Figure 1) and their respective decay characteristics, each peak was found to be slightly differently tailed, and hence, a simple restriction to shared tailing parameters, commonly applied in α-particle spectrometry, was found to lead to insufficiently well modeled tailing. To account for this, a special regression technique was developed that allows for differently tailed peaks, while maintaining reasonable convergence speed and robustness, considering the high number of required parameters. This is achieved by $\ell^{2}$-regularization of the tailing parameters, keeping them somewhat similar but not entirely shared among the peaks of each nuclide in the decay chain. Physically, this is motivated by the fact that the tailing is supposed to be similar, due to the relative similarity of the α-particle energies and the relatively small deviation in the effective path length through all absorbing layers. Specific details of this modeling procedure are given in Appendix A.

### 2.5. Estimation of ^222^Rn Emanation from Spectral Time-Series

As stated in Section 1.2, Equation (1), the evolution of the ^222^Rn activity retained in the IRSDs must follow first-order continuity, accounting for the emanation of ^222^Rn from the deposited layer, η, in terms of emanating ^222^Rn atoms per unit time.

From the analysis of IRSD spectrometric time-series, a discretized version of the potentially time varying ^222^Rn activity, $A_{Rn-222}\left(t\right)$, and the ^226^Ra activity, $A_{Ra-226}\left(t\right)$, are accessible through the analysis of the peak areas. The estimation of η (or derived quantities such as the emanation coefficient), based on those time-series, is an inverse problem unless a steady-state of $\frac{dA_{Rn-222}}{dt}=0$ has been reached. The observed version of $A_{Rn-222}\left(t\right)$ is given by the discretized convolution of η, with the impulse-response defined by the radioactive kinetics. In previous work [35,36], we developed and presented a deconvolution technique that allows the probability density function (PDF) of a discretized version of η(t) to be estimated, including the propagation of systematic uncertainty, using the supporting measurements of the residual ^222^Rn activity in the source.

This technique is based on recursive Bayesian inference, using a switching linear dynamical system model to estimate collections of multivariate PDFs for a state variable $x={\left[\begin{array}{cccc} A_{Rn-222} & A_{Ra-226} & \eta & \frac{\partial \eta }{\partial t} \end{array}\right]}^{T}$, given a collection of peak-areas derived from a collection of spectra $S_{1\ldots n}$ indexed by n, obtained at the measurement times $t_{n}$ in the set of all measurement times T, while $t_{N}$ describes the last measurement time instant. This includes inference of the filtering densities $p\left(x_{n}|S_{1\ldots n}\right)$, i.e., using, for each $x_{n}$, the information contained in all spectra, up to the time $t_{n}$, and their recursive correction into the smoothing densities $p\left(x_{n}|S_{1\ldots N}\right)$, using the information in all spectra, respectively. The algorithm accounts for potential changes of η during the integration time of the spectrum collection by integrating the forward propagation equations and using a certain Gaussian process autoregressive regularization on η with tunable parameters, which are optimized with respect to the marginal likelihood of the time-series. To improve fidelity during steep changes in η, as well as improving results in stable or slowly drifting regimes, the algorithm additionally estimates the probability for being in reasonably stable regimes along the time-series, with a process model for both the unstable and stable regimes. However, the uncertainty arising from the specifically chosen Gaussian process and their tuned parameters is not propagated, as this would require the evaluation of intractable integrals through computationally infeasible Markov–Chain Monte–Carlo and is considered negligible in comparison with the systematic uncertainty of the counting efficiency.

To estimate the IRSD emanation time-series in this work, this approach was modified to include the information on $A_{Ra-226}$ acquired in each spectrum, which was unused in the initial development for γ-ray spectrometry [35,36]. The input quantities to the algorithm, which, in the following, we refer to by the *SLDS-deconvolution*, are the peak-areas of ^222^Rn and ^226^Ra, the time-offset between each spectrum and their live times, a prior of the initial state, as well as an estimate of the PDF of the counting efficiency vector $\varepsilon =\left[\begin{array}{c} \varepsilon_{Rn-222}\\ \varepsilon_{Ra-226} \end{array}\right]$. Necessary inference equations are given in Appendix B, and for a detailed presentation of the algorithm, the reader is directed to [35,36]. The uncertainty in ε is propagated across the model using a sigma point method, as described in Appendix B. In steady-state situations, where $\frac{dA_{Rn-222}}{dt}=0$, η is simply given by the solution of Equation (1), using the components of the counting efficiency vector ε and the count rates of the respective nuclides as

(3) $$\eta =\frac{N_{Ra-226}}{\varepsilon_{Rn-226}t}-\frac{N_{Rn-222}}{\varepsilon_{Rn-222}t}$$

## 3. Results and Discussion

### 3.1. Morphological Characterization

**Figure 4:**
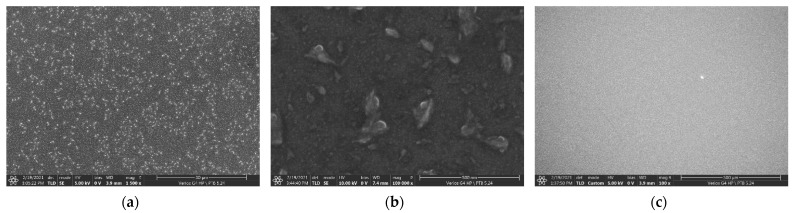
Secondary-electron SEM Images (through-lens detector) of thermal-PVD ^226^RaCl_2_ on a bare 1” prime Si-wafer at different magnifications: (**a**) 1500×, (**b**) 100,000×, (**c**) 100×. Length of bar: (**a**) 30 μm (**b**) 500 nm, (**c**) 500 μm.

The experimental procedure to construct the IRSD is demonstrated to form nano-crystalline deposits with typical particle sizes on the order of 10 nm and larger agglomerates of 100 nm, as shown in the SEM micrographs of a similarly manufactured modified Si-wafer (Figure 4a,b). At low magnification (Figure 4c), only a small number of larger impurities and defects can be identified. However, bright particles, of about 300 nm in size, in Figure 4a are most likely foreign particles, since they could also be observed on the shadowed portion of the wafer. The morphology of the surface appears to be considerably different than in our previously published work with electrodeposited ^226^Ra [16], where much more voluminous deposits were observed. Given that similar amounts of ^226^Ra were deposited in both studies, it is suggested that the present method produces considerably cleaner deposits.

However, since the layer is still visible to the naked eye at approx. 440 Bq (corresponding to approx. 15 ng of ^226^RaCl_2_, Figure 3a), most likely, it is composed almost entirely of other materials. This includes impurities present in the obtained ^226^RaCl_2_ solution, impurities introduced by all the solvents used, and impurities from the chamber materials at the respective deposition conditions. In the future, some of these impurities could potentially be avoided by a refined process, involving a mechanical shutter and feedback temperature control, to avoid contamination with readily volatile species. Nonetheless, the DSA α-particle setup showed a FWHM of only around 15 keV for the 4.78 MeV emission of ^226^Ra, where, previously, around 20 keV was measured for electrodeposited and around 16 keV for ion implanted ^226^Ra with the same setup [16,23], indicating relatively small α-particle energy loss within the deposited layer.

Figure 3a shows a photograph of a freshly prepared IRSD with approx. 440 Bq of ^226^RaCl_2_, hinting at the inhomogeneity of the deposit. This is more clearly evident in Figure 3b, a typical digital autoradiograph, obtained as explained in Section 2.3. The deposits of smaller activities, shown in Table 1, were initially not visible to the naked eye. However, they turned irreversibly slightly pale white upon exposure to very humid air. The deposits were found to be soluble in water, which means that IRSDs formed in this way should not be operated in condensing atmospheres.

The eccentrically peaked activity distribution is likely due to eccentric and/or non-perpendicular manual installation of the evaporation source. Nonetheless, the radiography image shows that effusion from the tantalum tube is quite directional, which is in line with our presumptions. Since part of the high deposition density area is shadowed by the aperture due to this eccentricity, the deposition efficiency could, potentially, be improved by optimization of the evaporator position and orientation.

### 3.2. Typical α-Particle Spectrum Features of the IRSD

Typically, and dependent on the characteristics of interfering electronic noise from the experimental setup, the FWHM of the higher energy ^226^Ra emission was found to be between 21 keV and 40 keV in the IRSD α-particle spectrum. While the observed FWHM are close to the manufacturer’s specifications (between 17 keV and 20 keV), pronounced low-energy tailing of the peaks can be identified that results from the high variability of the α-particle energy loss in the dead-layer (p-doped region) and deposited layers, as stated in Section 1.2.

**Figure 5:**
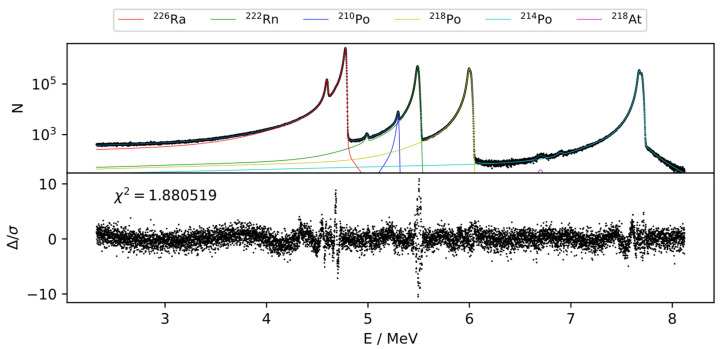
Typical α-particle spectrum obtained with a 442 Bq ^226^Ra IRSD (modified Canberra PIPS^®^ detector of nominal 100 μm depletion depth, 450 mm^2^ active area), over the first 6 · 10^5^ s after modification and regression results, according to Section 2.4.3. The 4.8 MeV ^226^Ra emission shows a FWHM of approx. 21 keV and a FWTM of approx. 48 keV. Progeny peaks appear slightly shifted to higher energies (wrt. the energy calibration obtained from the ^226^Ra emissions).

The deconvolution procedure, outlined in Section 2.4.3 and Appendix A, a typical result shown in Figure 5, yields that approximately 1.4%, 1.2%, 0.8%, and 0.5% of the total counts of the emissions related to the isotopes ^210^Po, ^222^Rn, ^218^Po, and ^214^Po, respectively, appear below a threshold of 4.8 MeV in the spectrum, i.e., the ^226^Ra region. Considering that, due to emanation, the amount of ^222^Rn and progeny present in the layers is around 50% of the amount of ^226^Ra, while ^210^Po is not present in significant quantities, this yields a maximum deviation of only 1.25% in the ^226^Ra area determination if the tailing contributions were entirely ignored.

In all recorded spectra, the tailing properties were found to be very similar, indicating that the main contribution to the energy loss is due to the dead-layer of similar thicknesses rather than due to the deposited layers of presumably variable areal densities.

Varying amounts of ^210^Po are also present. In general, the ^210^Po is introduced from ^210^Po in the original ^226^Ra solution, but it will also grow in slowly over time.

Remarkably, the ^214^Po and ^218^Po peaks of the spectrum show higher energy satellites with increasing energy shift between the main progeny peak and the satellite peaks in the order (^222^Rn) < ^218^Po < ^214^Po. An unresolved satellite peak might be present below the ^222^Rn peak, since this peak appears much broader than the main ^226^Ra emission. Due to the varying energy shift increasing along the decay chain, these satellites are thought to be related to the self-implantation of ^222^Rn and the short-lived progeny (SLP) ^218^Po and ^214^Po, as shown schematically in Figure 1. As a result, ^222^Rn and SLP are either present in the material deposited on the detector or injected into the p-doped region of the IRSD, due to their recoil, which is thought to cause the varying energy shift. In addition, some of the recoil energy might be detected in coincidence with the α-particle under some circumstances, i.e., injection of SLP to within the depletion zone. However, the energy shifts observed are on the order of 10 keV to 30 keV, while the coincidence of the α-particle, with the full recoil energy, would result in peak shifts of up to 200 keV. This indicates that ^218^Po and ^214^Po do not get implanted past the dead-layer to within the depletion zone.

Slight right-handed tailing of the ^226^Ra, ^222^Rn, and ^218^Po peaks can also be identified, attributable to random α-e and α-photon coincidences, while the usual pronounced right-handed tailing of the ^214^Po peak is due to the α-β true coincidence with the ^214^Bi β-particle, caused by the particularly small half-life of ^214^Po.

**Figure 6:**
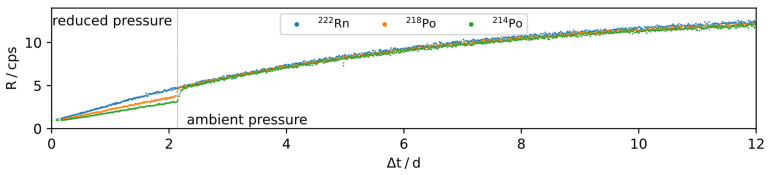
Ingrowth of α-particle count rates of an IRSD after initial ^226^Ra deposition at reduced and ambient pressure.

It was found that the ^214^Po and ^218^Po peak areas are smaller than the ^222^Rn peak area under all observed circumstances. The deviation of those two peaks from one another, and especially from the ^222^Rn peak area, was found to be dependent on the pressure of the environment as shown in Figure 6, depicting a time-series of the initial ingrowth of count-rates of the different nuclei in the deposited layer under reduced pressure and ambient conditions. Under ambient conditions, ^218^Po and ^214^Po count rates are observed to be approximately 1% to 2% lower than the ^222^Rn count rate. This can be explained by ^218^Po and ^214^Pb recoiling that leads to the additional ejection of ^218^Po and ^214^Pb, Figure 1. The mean free path of those nuclei in ambient pressure air is on the order of 100 nm, suppressing the recoiling strongly, where ^218^Po and ^214^Pb that lost enough energy, in proximity to the deposited layer, potentially remain adsorbed. However, some recoiling can still be observed, even in ambient pressure air. Due to this disturbance, with potential pressure sensitivity, the SLP peak areas were not further used to assess the activity of ^222^Rn remaining in the IRSD.

### 3.3. Efficiency Calibration

The counting efficiency of each IRSD was deduced from the data obtained through DSA α-particle spectrometry, Section 2.4, using the peak areas of ^226^Ra in each detector’s spectrum (DSA detector and IRSD), as determined by the regression models in Section 2.4.2. The counting efficiency of the IRSDs is hence given by

(4) $$\varepsilon_{Ra-226}=\frac{N_{\mathrm{Ra}-226,\;\mathrm{IRSD}}c_{\mathrm{Tail},\mathrm{IRSD}}}{N_{\mathrm{Ra}-226,\mathrm{DSA}}c_{\mathrm{backscatter}}c_{\mathrm{Tail},\mathrm{DSA}}}\frac{t_{\mathrm{DSA}}}{t_{\mathrm{IRSD}}}\frac{1}{4\pi }\frac{\int_{A}^{}w_{dA}\Omega_{dA}dA}{\int_{A}^{}w_{dA}dA}$$

where N describes the peak areas of the respective detector, t describes the live times of the respective detector, and *c* describes correction factors.

An overview of the obtained ^226^Ra counting efficiencies is given in Table 1, and the deposited ^226^Ra activity was determined analogously. Considering the uncertainty of this method, the counting efficiencies observed were generally very close to 0.5, with a maximum observed relative deviation of 1.6%. It is presumed that the deviation from the 2π sr geometrical efficiency, 0.5, results from backscattering and self-absorption losses in the deposited layer and the dead-layer of the IRSD. Since the covariance matrix estimate of the peak areas from the inverted Hessian of the regression procedure, outlined in Section 2.4.3, can lead to underestimation of the uncertainty due to non-stochastic residuals and amplification of numerical errors, and based upon the observations in Section 3.2, an additional normally distributed uncertainty, with a standard deviation of 0.3% for each peak area, was introduced to account for possible shortcomings of the modeling procedure. This value is based on the estimated tailing contributions of the short-lived progeny. As pointed out in earlier work, the contribution of scattered particles to the peak areas introduces an uncertainty of approximately 0.2% [16,37]. It is worthy to note that the backscattering causes an anti-correlation of the scattering bias of the DSA peak area and the IRSD peak area. However, this is intentionally not corrected to deduce the counting efficiency of the IRSD resulting from all effects. Due to layer areal density and random coincidence, the effective counting efficiency is expected to decrease with increasing ^226^Ra activity. However, in the investigated activity range this effect could not significantly be observed. Generally, uncertainty on the order of 1% in the counting efficiency or equivalently deposited activity was achieved using this method which is dominated by the uncertainty in the solid-angle resulting from the distance measurement of the recessed Si-surface of the IRSD relative to the standard geometry. An uncertainty budget is given as an example in Table 2 for the determination of the efficiency of the IRSD, with approx. 2 Bq ^226^Ra, where the least counting statistics were accumulated.

However, the DSA α-particle spectrometry does not allow for a precise measurement of the counting efficiency for ^222^Rn and SLP that resides within the IRSD, since the recoiling of ^222^Rn and SLP nuclei from the IRSD causes implantation into the geometrical components and the detector of the DSA setup. The emission of α-particles from those recoil implanted nuclides thus contributes to the peak areas of each detector to varying degrees. Due to this, no attempt was made to derive the ^222^Rn and progeny efficiency separately, and for the following, it is assumed that the ^222^Rn and ^226^Ra counting efficiencies are close to each other. Since the energy of the emitted α-particles of both nuclides are relatively similar, it is assumed that the backscattering and absorption losses are also similar. However, the recoiling causes slight displacement of the positional distribution of the ^222^Rn nuclei to the ^226^Ra positions, thereby possibly introducing a slight difference in the true counting efficiency. To model this effect, we assumed, in the following, that the counting efficiency of ^226^Ra and ^222^Rn is given by a multivariate normal distribution with a high correlation coefficient. However, for the following analyses using the counting efficiency, the peak-areas of ^222^Rn and ^226^Ra are thought to be anti-correlated, which has the opposite effect, and hence, a relatively balanced correlation coefficient of 0.6 was chosen to approximately model both effects.

(5) $$\left[\begin{array}{c} \varepsilon_{Rn-222}\\ \varepsilon_{Ra-226} \end{array}\right]\sim N\left(\mu_{\varepsilon_{Ra-226}}\left[\begin{array}{c} 1\\ 1 \end{array}\right],\sigma_{\varepsilon_{Ra-226}}^{2}\left[\begin{array}{cc} 1 & 0.6\\ 0.6 & 1 \end{array}\right]\right)$$

Regression model induced uncertainties can then be neglected, since those systematic factors are already included in the assumed counting efficiency distribution and the statistical uncertainty of the peak areas is estimated by assuming a Poisson distribution.

### 3.4. Estimation of ^222^Rn Emanation from IRSD Time-Series and its Humidity Dependence

As described in Section 2.5, the emanation of ^222^Rn in non-steady-state situations is estimated using the *SLDS-deconvolution* approach of the observed counts time-series, which is evaluated from the IRSD spectrometric time-series using the methods outlined in Section 2.4.3. The statistical uncertainty of the determined peak areas is estimated, recursively, alongside the prediction step of the filtering algorithm (Section 2.5, [36], Appendix B), accounting approximately for possible tailing contributions, as well as the Poisson statistics, as

(6) $$\Sigma_{n}\approx \varepsilon M\mu_{n-1}M^{T}\varepsilon^{T}+\sigma_{Area}2\varepsilon M\mu_{n-1}M^{T}\varepsilon^{T}\left[\begin{array}{cc} 1 & -0.8\\ -0.8 & 1 \end{array}\right]\varepsilon M\mu_{n-1}M^{T}\varepsilon^{T}$$

where $\mu_{n-1}$ is the mean of the state variable at the previous step, ε is the counting efficiency vector, M is a matrix that maps the state onto the measurement space, Appendix B, and $\sigma_{Area}$ is an additional uncertainty from uncertain area determinations, which was chosen according to the observations in 3.2 to be 0.005.

**Figure 7:**
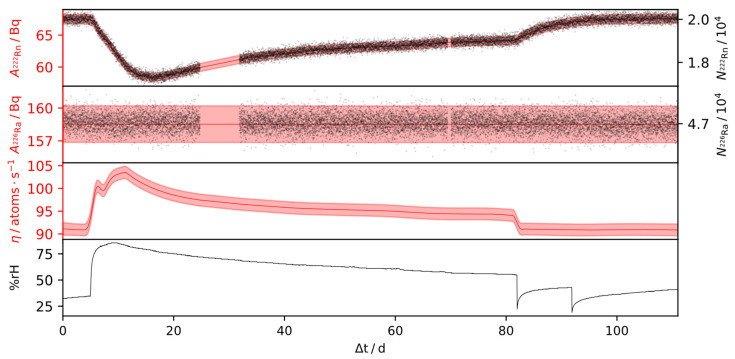
*SLDS-deconvolution* result for a IRSD of approx. 160 Bq ^226^Ra. Black dots represent the determined peak areas of a set of approx. 15,000 spectra, taken over 110 days at a sampling interval of 600 s of ^222^Rn and ^226^Ra, respectively. Red curves represent the smoothed results for the residual ^222^Rn- and ^226^Ra activities and the deconvolved time-series of the emanation η, according to Section 2.5. Shaded areas represent the 1σ credible intervals, almost entirely caused by the systematic uncertainty.

**Figure 8:**
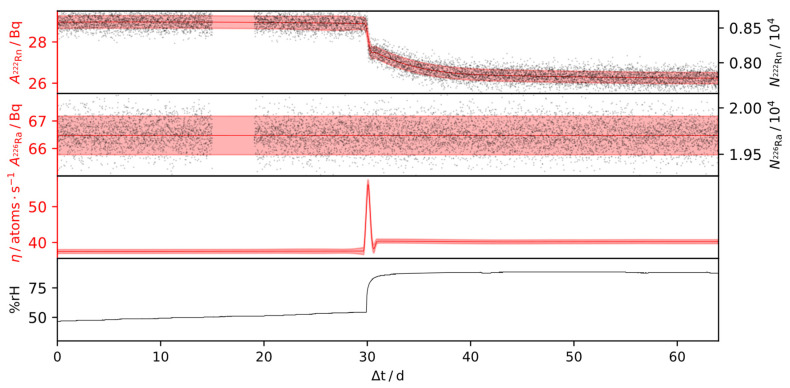
*SLDS-deconvolution* result for an IRSD of approx. 65 Bq ^226^Ra. Black dots represent the determined peak areas of a set of approx. 9000 spectra, taken over 65 days at a sampling interval of 600 s of ^222^Rn and ^226^Ra, respectively. Red curves represent the smoothed results for the residual ^222^Rn- and ^226^Ra activities and the deconvolved time-series of the emanation η, according to Section 2.5. Shaded areas represent the 1σ credible intervals, almost entirely caused by the systematic uncertainty.

Results of the outlined inference procedure for the IRSD, of approximately 160 Bq and 65 Bq, over time-series, consisting of 15,000 and 9000 α-particle spectra, are shown in Figure 7 and Figure 8, respectively. During these times, the relative humidity was varied in the range of 20% rH and 90% rH, while the temperature was kept constant. For the remaining IRSDs, experiments with dynamic conditions were not carried out and, therefore, the steady-state emanation is reported in Table 1, as the detectors showed stable emanation characteristics over 160 days (2 Bq detector) and 15 days (440 Bq detector) respectively, operating in a climate-controlled laboratory. Due to the high counting efficiency of the setup, the statistical uncertainty vanishes quickly alongside repeated observations within the time-series, in which case the combined relative uncertainty reduces to a steady value due to the systematic effects. Based on Equation (3), and using the observations from Section 3.3, the steady-state relative systematic uncertainty of η can thus be expressed by linearization as

(7) $$\frac{\sigma_{\eta }}{\eta }=\frac{\sigma_{\varepsilon }}{\varepsilon }\sqrt{1+2\frac{N_{Ra}N_{Rn}}{{\left(N_{Ra}-N_{Rn}\right)}^{2}}\left(1-\rho_{\varepsilon }\right)}\;$$

where $\rho_{\varepsilon }$ denotes the correlation coefficient of $\varepsilon_{Rn-222}$ and $\varepsilon_{Ra-226}$.

Note that the relative uncertainty in η decreases with an increasing correlation coefficient of the counting efficiency vector. Based on the proposition of a counting efficiency correlation coefficient of 0.6 and the typically observed 50% emanation, this evaluates to approximately 1.5% to 2% systematic uncertainty in η, depending on its absolute value and the relative uncertainty of the counting efficiency. Based on this observation, the sigma point method outlined in Appendix B proves to be the best option for uncertainty propagation in the *SLDS-deconvolution*.

From the presented deconvolution results in Figure 7 and Figure 8, it is evident that the relative humidity impacts the emanation positively. Therein, changes of the steady-state values were observed up to 15% on a range between 20% rH and 90% rH. However, the humidity dependence shows non-linear behavior, with an increasing rate of change at higher relative humidity values. Interestingly, peaks in emanation can be observed in response to steep positive gradients in the humidity, while jump changes from high to low humidity do not cause those peaks. Therefore, the behavior of the IRSD depends on the direction of a change in the ambient relative humidity, i.e., whether the deposit is wetting or drying.

The explanation for this effect lies in the state of disequilibrium that results from each of those changes. While most of the ^222^Rn retained within the IRSD is buried in the silicon wafer, such as in Figure 1, some of it is evidently loosely adsorbed to the surface or within the ^226^RaCl_2_ layer. Those ^222^Rn nuclei can evidently be desorbed in response to a change in relative humidity (Figure 7 10 d and Figure 8 30 d), such that the activity of retained ^222^Rn quickly drops, resulting in a peak in the emanation. If the release of those ^222^Rn nuclei, however, is not enough to reach the equilibrium point, a characteristic decay period of the retained ^222^Rn nuclei, with the kinetics of the radioactive decay, follows. Conversely, during a change from high to low emanation, the only way for the activity of retained ^222^Rn to reach equilibrium is the ingrowth from the ^226^Ra decay, since back-diffusion evidently does not occur (Figure 7, >80 d), which follows a characteristic ingrowth curve. Additionally, the change of the effective emanation conditions can follow its own dynamics, as during the initial rise in Figure 7. Given that the rate of re-equilibration of the device depends on the state it was previously in, hysteresis may occur. Nonetheless, it is shown in Figure 7 that the emanation from the IRSD recovers its initial value after exposure to more humid air.

These observations underpin the propositions of Section 2.5 and those given in [35,36].

## 4. Conclusions

In this work, we have demonstrated the successful development of a novel device, in conjunction with analytical procedures, to standardize the emanation of ^222^Rn from a solid source of ^226^Ra. For the first time, a complete approach based on a unique combination of a ^222^Rn source with a spectrometric detector, the IRSD, was presented. This allows the continuous monitoring of the residual amount of ^222^Rn in the source by highly efficient, direct α-particle measurements under ambient conditions, which drastically improves the reliability under changing operational characteristics and environmental parameters. Additionally, the direct measurement of the α-particles, emitted by the residual ^222^Rn, specifically enabled by the design of the IRSD, alleviates some of the sources for systematic uncertainty of previous γ-ray spectrometry based approaches [16,18] while providing a means of achieving unmatched counting efficiency at a negligible background. As was demonstrated, ^222^Rn is not necessarily in secular equilibrium with the short-lived progeny in a thin-layer, which could result in a bias of approximately 2% if γ-spectrometry of ^214^Pb and ^214^Bi was applied as a monitoring tool instead.

The very high counting efficiency of the IRSD technique allows to achieve high statistical accuracy in short integration times and, thereby, leads to improved performance of procedures to infer the emanation. The small sampling interval allowed for identifying even relatively narrow peaks in the emanations that result from increases in the relative humidity of the environment. The continuous reliable measurement of these considerably small ^222^Rn emanation terms (corresponding to a single ^222^Rn atom released per second) allows refining calibration procedures in the future, e.g., by down-scaling of reference volumes, injection of collected amounts of ^222^Rn directly into measurement systems, reductions of flow rates, and other, similar methods. Thereby, the definition of the unit Bq⋅m^−3^ can be realized by integrating the radioactive decay kinetics, driven by the derived emanation source terms ranging from 2 µBq⋅s^−1^ to 440 µBq⋅s^−1^, with a combined uncertainty not exceeding 2% and thus, providing one of the most advanced techniques of low-level ^222^Rn standardization to this end. The application of the IRSD setup thus allows one to realize and disseminate reference atmospheres of ^222^Rn in the ambient concentration range, traceable to the SI.

It is presumed that this range can be readily extended in both directions since the statistical uncertainties were low, even in the low ^226^Ra activity IRSD, while the higher activity ones did not begin to show significant losses from random coincidence and self-absorption.

A possible pathway of improvement of the uncertainty is a refined distance measurement in the defined-solid angle α-particle spectrometry, which was the overall main contributor to the uncertainty of the determined counting efficiency. During the area determinations of the α-particle spectra, a heuristic approach, to estimate the uncertainty arising from the tailing contributions, was applied. This was found necessary due to computational limitations, but it can be improved on once more advanced techniques for deconvolution of α-particle spectra become available. In a similar vein, future improvements in modeling techniques for time-series, similar to those applied in Section 2.5 and Appendix B, as well as increases in available computational resources, will translate to even more realistic uncertainty estimation, potentially allowing us to drop some of the assumptions made.

While it was possible to investigate the behavior of the IRSD setups over approximately 1 year in total, it is expected that certain characteristics of the IRSD will degrade over time, due to the continuous self-implantation of nuclei, leading to potential knock-ons in the p-doped dead-layer of the detector, and thus, to a degradation of its junction properties, especially concerning higher activities of ^226^Ra than those used in this study. Moreover, the ^210^Po peak is a disturbance in the spectra that were collected, and hence, the ingrowth of ^210^Po will additionally degrade the information that can be inferred from the spectra or, at least, increase the uncertainty of the determined emanation. Due to the relatively long half-life of intermediate ^210^Pb, however, this process takes place on the scale of tens of years. Currently, the technique is only applied at PTB, and this first presentation of it serves as a proof of principle and to lay down the traceability chain to the SI. However, continuous production of IRSD of different activity, for dissemination and potential future replacements, by the PTB is limited to a low volume of devices. Due to the relative simplicity of the process to create an IRSD, using relatively low-cost and rugged components, supply of such setups can, potentially, be realized in the future by an implementation in industry to disseminate the unit Bq⋅m^−3^, concerning ^222^Rn in the air, in the presented way.

## Figures and Tables

**Table 1:** Overview over produced IRSD.

Detector Type	Active Area/Depletion Depth	A(^226^Ra)/Bq	$\varepsilon_{Ra-226}/\mathrm{cps}\;\mathrm{Bq}{-}^{1}$	Observed Mean η
Mirion PIPS^®^	450 mm^2^/300 μm	1.91 ± 0.02	0.502 ± 0.006	0.999 ± 0.017
Ametek Ortec ULTRA^®^	450 mm^2^/300 μm	66.4 ± 0.5	0.494 ± 0.004	Figure 8
Mirion PIPS^®^	450 mm^2^/300 μm	158.6 ± 1.7	0.494 ± 0.005	Figure 7
Mirion PIPS^®^	450 mm^2^/100 μm	442 ± 4	0.492 ± 0.005	209 ± 4

/ represents division by a unit.

**Table 2:** Example uncertainty budget of efficiency determination of an IRSD of 2 Bq ^226^Ra.

Description and Type	Value and Uncertainty	Rel. Uncertainty	Rel. Contribution
Solid angle (systematic)	(0.00940 ± 0.00006) 4π sr	0.6%	28.4%
Backscattering_DSA_ (systematic)	1 ± 0.002	0.2%	3%
Tailing_DSA_ (systematic)	1 ± 0.003	0.3%	6.7%
Tailing_Si_ (systematic)	1 ± 0.003	0.3%	6.7%
^226^Ra rate_DSA_ (stochastic)	$(0.01796\;\pm \;0.00015)\;s^{-1}$	0.8%	55.1%
^226^Ra rate_Si_ (stochastic)	$(0.9595\;\pm \;0.0004)\;s^{-1}$	0.04%	0.1%
$\varepsilon_{Ra-226}$	0.502 ± 0.006	1.2%	

## Data Availability

Collected spectrometric data, as well as the custom software for regression analysis and statistical inversion is available on request from the authors.

## Appendix A

The models for peak shapes in α-particle spectra described in [29,30] were further refined in this work to provide improved numerical stability, better convergence properties, and more physically reasonable results, as outlined below.

A numerically stable equivalent representation of this peak shape is given by Equation (A1) through a piecewise definition in terms of the scaled complementary error function *erfcx* (erfcx(x) = exp(x^2^)erfc(x)), which was computed by Chebyshev approximation [38]. Flipping the sign on x and μ, Equation (A1) is similarly used to represent right tailed Gaussians.

(A1) $$f_{i}\left(x,\mu_{i},\sigma_{i},\tau_{i}\right)=\{\begin{array}{c} \frac{1}{2}\tau_{i}\exp\left(a_{i}\right)\mathrm{erfc}\left(b_{i}\right)\;\forall \;a_{i}\leq 0\\ \frac{1}{2}\tau_{i}\exp\left(c_{i}\right)\mathrm{erfcx}\left(b_{i}\right)\;\forall \;a_{i}>0 \end{array}\;\mathrm{where}\;a_{i}=\left(x-\mu_{i}\right)\tau_{i}+\frac{1}{2}\sigma_{i}^{2}\tau_{i}^{2},\;b_{i}=\frac{1}{\sqrt{2}}\left(\frac{x-\mu_{i}}{\sigma_{i}}+\sigma_{i}\tau_{i}\right),\;\;\mathrm{and}\;c_{i}=-\frac{{\left(x-\mu_{i}\right)}^{2}}{2\sigma_{i}^{2}}$$

Each peak in the spectrum is represented as a weighted sum over different realizations of Equation (A2), which represents the probability density function of a mixture of exponentially modified Gaussians (ExGaussian).

(A2) $$P_{n}\left(x,w_{n},\mu_{n},\sigma_{n},\tau_{n}\right)=\overset{k}{\underset{i=0}{\sum }}w_{n,i}f_{i}\left(x,\mu_{n,i},\sigma_{n,i},\tau_{n,i}\right)$$

where n represents the peak index and i represents the respective tail index. $w_{n}$, $\mu_{n}$, $\sigma_{n}$, and $\tau_{n}$ represent the corresponding vectors of shape parameters, indexed by i.

The *softplus* transformation was used to constrain the parameters to positive values generally, except for the weights $w_{n,i}$. The k components of the weights vectors $w_{n}$ are generally restricted as compositional factors on the k−1 dimensional simplex and are thus transformed to a k−1 component vector using an *isometric log-ratio* transformation [39], such that the components of $w_{n}$ are constrained within (0,1) and sum to 1.

The modeled spectrum $\hat{S}$ can then be written in Matrix notation, where ***A*** is a vector containing the peak-areas and R is a matrix made up of the peak-shapes as the column vectors. The modeled spectrum is thus given by the linear-combination of the column-vectors of R,

(A3) $$\hat{S}=RA$$

For a given R, and a measured spectrum S of l chosen regression bins and of high statistical accuracy (i.e., where the spectrum can reasonably be assumed to follow a multivariate Gaussian with covariance matrix diag(S)), ***A*** is given as the solution to the weighted, linear least-squares problem

(A4) $$\left(R^{T}OR\right)A=R^{T}OS$$

where $O=\mathrm{diag}\left(S^{-1}\right)$ denotes the diagonal matrix of bin-wise reciprocals of ***S***.

This linear sub-problem is efficiently solved by Cholesky decomposition.

To determine the shape-parameters stacked in a parameter vector θ using a spectrum of high statistical accuracy, the following regularized objective is used, splitting the optimization into a linear and a non-linear sub-problem and implicitly defining A, where f(θ) is a function that regularizes the peak-shape parameters, as explained below.

(A5) $$ℒ=\frac{1}{2l}{\left(S-\hat{S}\right)}^{T}O\left(S-\hat{S}\right)+f\left(\theta \right)==\frac{1}{2l}{\left(S-R{\left(R^{T}OR\right)}^{-1}R^{T}OS\right)}^{T}O\left(S-R{\left(R^{T}OR\right)}^{-1}R^{T}OS\right)+f\left(\theta \right)$$

θ is optimized by minimizing ℒ using the quasi-Newton Broyden–Fletcher–Goldfarb–Shanno (BFGS) minimization procedure as implemented in the Python package SciPy [40]. The required gradient $\frac{\partial ℒ}{\partial \theta }$ is computed exactly by automatic reverse-mode differentiation using the Jax Python framework [41] and custom differentiation rules for Equation (A1).

It is not physically sensible that the peak shapes vary drastically between the different nuclides in the decay chain, apart from the position of the mode. However, due to displacement from recoiling further in the decay chain, and possibly due to α-e and α-photon true coincidence effects, slight differences exist between the peak shapes, which must be addressed in the modeling procedure. For this reason, the shape parameters are not shared across the different peaks resulting in many degrees of freedom. Consequently, those models are strongly dependent on the starting parameters without regularization since the likelihood space is thought to have many local maxima. As a result it is common for flat regions in the spectrum to be erroneously attributed to a single peak while the tailing of the others is strongly underestimated. In this work, a penalty term *f*(θ) is used to address this observation by capturing the physics of the spectrum to improve the convergence into a physically sensible minimum, i.e., where the peak shapes are similar, but not completely shared. *f*(θ) regularizes the w- and τ-vectors of each peak, such that they are closely related amongst chosen peaks by a $\ell^{2}$-penalty,

(A6) $$f\left(\theta \right)=\frac{1}{2}\overset{j}{\underset{n=1}{\sum }}{\left({\hat{w}}_{0}-{\hat{w}}_{n}\right)}^{T}{\left(\kappa {\hat{w}}_{0}{}^{2}\right)}^{-1}\left({\hat{w}}_{0}-{\hat{w}}_{n}\right)+{\left(\tau_{0}-\tau_{n}\right)}^{T}{\left(\gamma \tau_{0}{}^{2}\right)}^{-1}\left(\tau_{0}-\tau_{n}\right)$$

where ${\hat{w}}_{n}$ describes the n-th transformed weight vector, $\tau_{n}$ is the *n*-th tailing vector, *j* is the total number of regularized peaks, κ and γ are parameters that control the strength of the regularization. κ=γ=0.04 was found to provide reasonable regularization, judging from unregularized reduced $\chi^{2}$ values. Specific values of $\tau_{i,n}$ and ${\hat{w}}_{i,n}$ (e.g., these corresponding to the ^214^Po alpha-beta true coincidence region, Figure 5.) are excluded from the regularization function to obtain better results. Due to true coincidence effects already pointed out in earlier work on ^226^Ra α-particle spectrometry [42], the right-handed tails of the lower energy ^226^Ra emission were also excluded from the regularization procedure due to the pronounced α-e true coincidence resulting from the high counting efficiency and the highly converted levels in the decay of ^226^Ra [42]. For each peak, a total of 6 left-handed and 2 right-handed tailing terms were used, which provided good deconvolution results.

It should be noted that the penalization acts on the transformed weights but the untransformed tailing parameters. Due to the *softplus* transformation of each τ and the regularization acting on the untransformed value, this regularizes strongly tailed contributions much more than weakly tailed contributions, which penalizes physically unmeaningful results, i.e., those where low-energy tailings of the peaks are strongly dissimilar. Moreover, when fitting peak-shapes given by Equation (A2), the peak areas are commonly the parameters with the smallest gradient of the objective function. The splitting of the linear sub-problem additionally reduces the sensitivity towards the starting parameters, as, for each **R**, the best fitting ***A*** is implicitly found during each iteration of the chosen non-linear optimization procedure.

In principle, an asymptotic estimate of the covariance matrix of A can be found by truncated Taylor expansion, assuming it follows a multivariate normal distribution and residuals are purely stochastic, as

(A7) $$\Sigma_{A}\approx \frac{1}{l}\left(\frac{\partial A}{\partial \theta }\right){\left(\frac{\partial^{2}ℒ}{\partial \theta^{T}\partial \theta }\right)}^{-1}{\left(\frac{\partial A}{\partial \theta }\right)}^{T}$$

which was generally found to provide unreasonable results due to numerical issues from bad conditioning of the Hessian. The uncertainty is thus instead estimated from heuristic approaches and the underlying Poisson statistics, as outlined in the respective sections of the results.

To determine the peak-areas in spectra of low statistical accuracy (e.g., for the area determinations of spectrometric time-series with small integration times), the shape parameters as deduced from a spectrum of similar shape and high statistical accuracy are held constant to define the Matrix R and only the vector A is determined for each spectrum at hand. However, obtaining A by solving Equation (A4) as the least-squares solution leads to biased results in this case due to the Poisson statistics being much more relevant in cases where a lower number of counts is observed in each bin. In some cases, the bias experienced in this way is as high as 5%, which was found by comparing the least-squares solutions to the asymptotic values. Therefore, the following alternative maximum likelihood formulation was used, where A is determined by minimizing the negative log-likelihood under Poisson distributed counts in each channel and for each spectrum for the given **R** matrix,

(A8) $$ℒ\propto \underset{i}{\sum }{\left(RA\right)}_{i}-S_{i}\ln{\left(RA\right)}_{i}$$

which can not be solved in closed-form, and hence, BFGS is used.

This overall approach allows for a reliable and reasonably fast determination of peak areas in spectra with low statistical accuracy (e.g., in the time-series), since the peak shapes are already determined independently, allowing for better estimation of the tailing contributions. This allows for smaller integration times to be chosen during the collection of time-series and thus a higher temporal resolution.

## Appendix B

The filtering and smoothing algorithm denoted by *SLDS-deconvolution* in this work is based on modeling the emanation η as a Gaussian process, such that the state vector x follows the following Itō stochastic differential equation, as reported in [36],

(A9) $$dx=Kxdt+L{d\beta }_{t}\;x_{s}={\left[\begin{array}{c} A_{\mathrm{Rn}-222}^{S}\\ A_{\mathrm{Ra}-226}^{S}\\ \eta \\ \frac{d\eta }{dt} \end{array}\right]}_{s};\;K_{s}=\left[\begin{array}{cccc} -\lambda_{\mathrm{Rn}-222} & \lambda_{\mathrm{Rn}-222} & -\lambda_{\mathrm{Rn}-222} & 0\\ 0 & \lambda_{\mathrm{Ra}-226} & 0 & 0\\ 0 & 0 & 0 & 1\\ 0 & 0 & 0 & -\gamma_{s} \end{array}\right];\;L_{s}=\left[\begin{array}{c} 0\\ 0\\ 0\\ \sigma_{s} \end{array}\right]$$

where $\gamma_{s}$ and $\sigma_{s}$ denote optimizable parameters and the index s corresponds to the active system model.

In the applied switching linear dynamical approach, multiple linear dynamical models indexed by s operate by a method analogous to the classical Kalman-Filter [43] and their results are merged into a common PDF according to the likelihood of their prediction. Smoothing is carried out directly using the approach in [44], as reported in [36]. In practice, 2 models with distinct $\sigma_{s}$ but shared γ are used to provide better results.

The measurements y (which are the vectors of peak areas in the present case) are modeled to be related to the evolution of the state variable by,

(A10) $$y\left(t,r\right)=\varepsilon \int_{0}^{r}x\left(t+\tau \right)d\tau$$

such that the joint distribution of measurement and state can be expressed as [36],

(A11) $$p\left(x_{n},y_{n}|y_{1:n-1}\right)\propto N\left(\left[\begin{array}{c} F_{r}F_{\delta }\mu_{n-1}\\ HMF_{\delta }\mu_{n-1} \end{array}\right],\left[\begin{array}{cc} F_{r}\left(F_{\delta }\Sigma_{n-1}F_{\delta }^{T}+U_{\delta }\right)F_{r}^{T}+U_{r} & HM\left(F_{\delta }\Sigma_{n-1}F_{\delta }^{T}+U_{\delta }\right)F_{r}^{T}+HC^{T}\\ F_{r}\left(F_{\delta }\Sigma_{n-1}F_{\delta }^{T}+U_{\delta }\right)M^{T}H^{T}+CH^{T} & HM\left(F_{\delta }\Sigma_{n-1}F_{\delta }^{T}+U_{\delta }\right)M^{T}H^{T}+HBH^{T}+\Sigma \end{array}\right]\right)$$

$F_{a}=e^{Ka}$	$U_{a}=\int_{0}^{a}e^{K\left(a-\tau \right)}LL^{T}e^{K^{T}\left(a-\tau \right)}d\tau$
$M_{r}=\int_{0}^{r}e^{K\tau }d\tau$	$C_{r}=\int_{0}^{r}\int_{\tau }^{r}e^{K\left(r-\tau \right)}LL^{T}e^{K^{T}\left(a-\tau \right)}\;da\;d\tau$
$B_{r}=\int_{0}^{r}\int_{\tau }^{r}\int_{\tau }^{r}e^{K\left(a-\tau \right)}LL^{T}e^{K^{T}\left(b-\tau \right)}\;da\;db\;d\tau$	

where *r* denotes the integration time of the spectrometer. This density, recursively evaluated and conditioned on the measurements, is the basis of the filtering procedure and the obtained filtering PDF is recursively corrected into a smoothed posterior PDF conditioned onto the whole time-series.

Due to the Poisson noise of the measurements, and following the propositions in [36,45] as well as approximately including the correlated variance of the peak areas due to tailing, the measurement noise covariance is estimated in each filtering step from the previous state estimate mean as

(A12) $$\Sigma \approx \varepsilon M\mu_{n-1}M^{T}\varepsilon^{T}+\sigma_{\mathrm{Area}}2\varepsilon Mx_{n-1}M^{T}\varepsilon^{T}\left[\begin{array}{cc} 1 & -0.8\\ -0.8 & 1 \end{array}\right]\varepsilon M\mu_{n-1}M^{T}\varepsilon^{T}$$

Uncertainty propagation considering the counting efficiency vector ε is given by Equation (A13), which is intractable. The uncertainty of the counting efficiency is approximately propagated across the *SLDS-deconvolution* model by evaluating the model at a select set of *sigma points* in p(ε) and computation of the enveloping multivariate Gaussian for each of the computed $p\left(x_{n}|y_{1\ldots n},\varepsilon \right)$ and $p\left(x_{n}|y_{1\ldots N},\varepsilon \right)$.

(A13) $$p\left(x_{n}|y_{1\ldots n}\right)=\int^{\;}p\left(x_{n}|y_{1\ldots n},\varepsilon \right)p\left(\varepsilon \right)d\varepsilon$$
